# Biologics in COVID-19 So Far: Systematic Review

**DOI:** 10.3390/ph15070783

**Published:** 2022-06-23

**Authors:** Milton Arias, Henry Oliveros, Sharon Lechtig, Rosa-Helena Bustos

**Affiliations:** 1 Department of Clinical Pharmacology, Evidence-Based Therapeutics Group, Faculty of Medicine, Universidad de La Sabana and Clínica Universidad de La Sabana, Autopista Norte de Bogotá, Chía 140013, Colombia; milton.arias@unisabana.edu.co (M.A.); sharonlechtig@hotmail.com (S.L.); 2 Department of Epidemiology, Health Research Group, Faculty of Medicine, Universidad de La Sabana, Campus del Puente del Común, Km. 7, Autopista Norte de Bogotá, Chía 140013, Colombia; henry.oliveros@unisabana.edu.co

**Keywords:** SARS-CoV-2, COVID-19, biologics, Biopharmaceuticals, Interleukin inhibitors, Interferon treatment, mesenchymal stem cells, anti-spike monoclonal antibody

## Abstract

This systematic review aimed to reevaluate the available evidence of the use of biologics as treatment candidates for the treatment of severe and advanced COVID-19 disease; what are the rationale for their use, which are the most studied, and what kind of efficacy measures are described? A search through Cochrane, Embase, Pubmed, Medline, medrxiv.org, and Google scholar was performed on the use of biologic interventions in COVID-19/SARS-CoV-2 infection, viral pneumonia, and sepsis, until 11 January 2022. Throughout the research, we identified 4821 records, of which 90 were selected for qualitative analysis. Amongst the results, we identified five popular targets of use: IL6 and IL1 inhibitors, interferons, mesenchymal stem cells treatment, and anti-spike antibodies. None of them offered conclusive evidence of their efficacy with consistency and statistical significance except for some studies with anti-spike antibodies; however, Il6 and IL1 inhibitors as well as interferons show encouraging data in terms of increased survival and favorable clinical course that require further studies with better methodology standardization.

## 1. Introduction

At the end of 2019, a cluster of patients with pneumonia were identified in the city of Wuhan in the Hubei province in China. The behavior of the disease resulted in a fast-spreading epidemic throughout the country. In the initial approach to etiological mechanisms a hypothesis of zoonotic transmission was established, however, in a short period of time, sustained human-to-human transmission is confirmed, leading to the recognition that the disease had pandemic potential [1]. In February of 2020, the world health organization (WHO), acknowledges the disease as “coronavirus disease 2019” (COVID-19) and the virus that is purified as an etiologic agent as “severe acute respiratory syndrome coronavirus 2” (SARS-CoV-2), given that its genomic sequence is related to the virus responsible for the SARS outbreak in 2003 [2].

This pandemic has claimed over 4.7 million fatalities since its beginning and although the trend shows a progressive decline in new cases and deaths, the situation is still serious with over 3.3 million new cases and over 55,000 new deaths reported in the second half of September 2021, according to the WHO weekly reports [3]. This proportion of cases/deaths are related to the fact that COVID-19 mostly presents itself as a self-limited mild respiratory disease (80% of cases) [4], yet the cumulative number of affected patients determine a large mortality. There is no doubt that there is a need to treat the late stages of the disease, beyond the focus of vaccination as a preventive measure. Amongst the candidates undergoing scrutiny, it is desirable to address the ones with the most specific targets possible in order to avoid severe adverse effects in patients who are already in a situation with a considerable mortality rate. Accordingly, this article will aim to review the available studies regarding the use of biologics in COVID-19 critically or severely ill patients. Biologic medicines are called biologics and can be created using biotechnology or other cutting-edge technologies [5]. The final rule modifies the FDA regulatory definition of “biologic product” to include “any alpha amino acid polymer with a specific defined sequence that is greater than 40 amino acids in size” [6]. Although also biological, they are composed of sugar, proteins, nucleic acids, or complex combinations of these substances. Thus, biologicals include blood components, recombinant proteins, vaccines, monoclonal antibodies, and gene therapy, among others [7].

First, we must briefly review the comprehension of the common pathophysiological model to understand the reasons behind the selected drugs along the review. In the case of COVID-19 disease, three clinical phases can be categorized according to severity: onset of the disease, pulmonary phase, and hyper inflammation phase.

The first stage of the disease is usually characterized by mild symptoms similar to those of seasonal influenza [2]. In this stage, it is considered that the virus contacts the respiratory epithelial tissues as a predilection site of entry. Concerning this phenomenon, the first contact mechanism between the virion and the cell is through the viral crown. The virus has certain structural proteins called “spikes” that protrude from the membrane of the virion; this gives it the characteristic appearance of a crown in electron microscopy, which is reflected in the name of the virus. These spikes are transmembrane trimeric glycoproteins that are composed of two functional subunits, S1 and S2 [8,9]. It is these glycoproteins that determine the diversity of coronaviruses in terms of the tropism towards their hosts and specific tissues in an organism.

SARS-CoV-2 in this aspect shows an affinity for angiotensin-converting enzyme 2 (ACE 2) using it as a functional receptor, however, it is not the only mediator involved in the binding of the virus to the host cell. In the most recognized model, the way of entry of the virus is through endocytosis. Once inside, the virion, must fuse its membrane with the endosome, and thus release its RNA; for this purpose, it uses the transmembrane protease serine 2 (TMPS2) or the L-cathepsin that cleaves the spike into the S1 and S2 subunits. The S1 subunit ensures the stability of the anchorage to the membrane, whereas the splitting of S2 requires a second cleavage at S2 to generate a conformational change to consolidate the fusion [2,9].

Although this is the most accepted model, in fact, the particularity of this virus compared to other coronaviruses is the type of cleavage sequence “reverse-phase protein array (RPPA)” at the S1/S2 site, which is susceptible to furin [10]. Considering the ubiquity of furin, it is not surprising that this virus is highly pathogenic. Although its tropism for the angiotensin-converting enzyme explains its ease of entry through respiratory epithelia, heart, ileum, kidney, and bladder [11], its ability to compromise in other systems and its impact on the reticuloendothelial system may have to do with its RPPA cleavage sequence.

Once inside, the virus must proceed to make use of the nuclear and ribosomal machinery to achieve the replication of its RNA and biosynthesis of structural and non-structural proteins. Considering that the structural components correspond to the membrane, envelope, nucleocapsid, and spikes, non-structural proteins and their interaction with the cellular machinery are of interest as possible therapeutic targets. The evidence regarding cell interactions is extrapolated from the lessons learned in the study of SARS and MERS as close relatives of SARS-CoV-2. In this sense, it is derived that the RNA of our coronavirus consists of 11 open reading frames, which encode 16 non-structural proteins (NSP) that encompass most of the mechanisms implied in the pulmonary phase [12,13,14,15,16,17,18,19,20]. Considering this fact, we will not expand in the function of each NSP and will proceed with the characteristic phase of the critically ill, the hyper inflammation phase.

The hyper inflammation phase axis is the interaction of the virus with the immune system; the primary contact to establish is with the innate immune system. In this category, the pulmonary epithelium mainly has macrophages, which can appear in the apical epithelium, and also dendritic cells are usually found in the sub-epithelium. The immediate predictable consequence is the phagocytosis of apoptotic epithelial cells extrapolating models related to influenza viruses [21]. Koichi Yuki et al. suggest another kind of approach to this issue, implying that the coronavirus has the potential for direct infection in dendritic cells by replacing its ACE2 receptor with the specific adhesion molecule of dendritic cells (non-integrin trapping molecule 3) [9]. The chain of events continues with the presentation of the pathogen to the T cells of the immune system; this event results in the release of chemotactic that promotes the massive recruitment of other lymphocytes. It is possible that the lymphopenia observed in patients with hyperinflammation is related to this fact [22,23].

The presence of multiple inflammatory cytokines has been identified in severely ill COVID-19 patients. Interleukin 1 (IL)-1, IL-6, IL-10, granulocyte colony-stimulating factor (GCSF), monocytic chemoattractant protein 1 (MQP1), macrophage inflammatory protein (MIP) 1α, and tumor necrosis factor (TNF)-α are relevant [22,23,24]. In the study by Yonggang Zhou et al, both the cytokine storm and the distribution of the lymphocyte subpopulations, or at least the expression of the flow cytometry, are evaluated. The presence of CD69, CD38, and CD44 are highlighted, demonstrating the recruitment of both T CD8^+^ and T CD4^+^. In turn, it is worth noting the increased expression of control receptors Tm3 and PD-1 in both subpopulations of T cells, displaying depletion of cell populations [23]. It can be suggested that lymphocyte depletion may perpetuate a poor immune response to the pathogen; all this is favored by the mentioned cytokine storm microenvironment. Lymphocytic infiltration and the depletion of T cells is not the only problem that occurs in this microenvironment; it has been reported that, in patients with severe lung injury, there is a correlation with the cellular population predominance of macrophages and neutrophils in the pulmonary epithelium. [25]. To achieve this phenomenon, the immune response must use both interferon (IFN) γ and granulocytic-macrophage colony-stimulating factor (GMCEF). In this scenario, the host uses abnormal CD4 T cells that express both mediators [23].

In this review, we will aim to describe the use of biological drugs in adult patients with confirmed SARS-CoV-2 infection, preferring severe or advanced stage of compromise and their efficacy in clinical practice according to the available evidence so far.

## 2. Results

**Figure 1:**
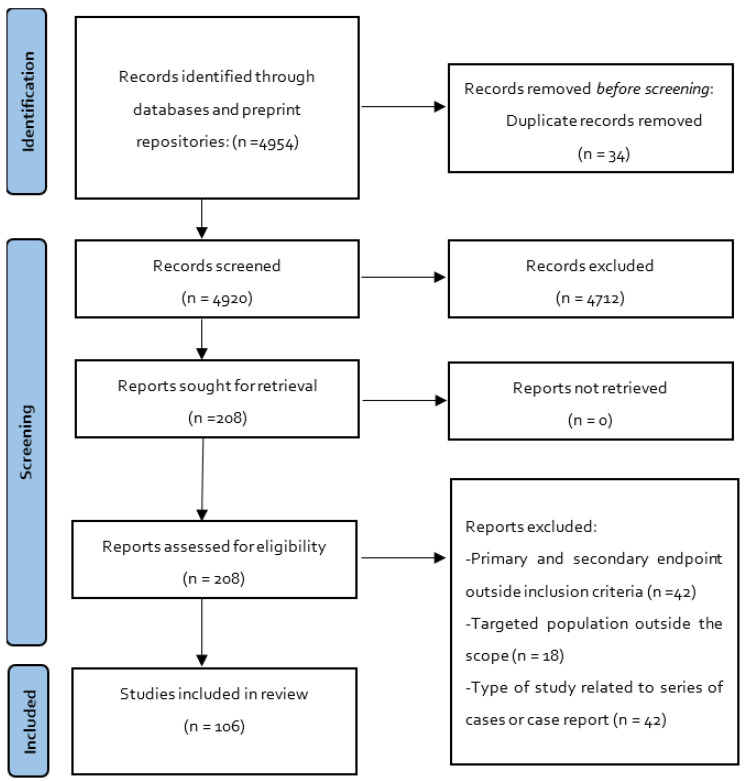
Flowchart of selected studies.

The process of selection and the number of articles selected was performed as de-scribed in the following chart (Figure 1). Table 1 shows the biotherapeutics in COVID-19 patients.

### 2.1. Interleukin 6 Inhibitors

Interleukin 6 (IL-6) is one of the most popular targets regarding the abundance of evidence generated since 2020. It is comprehensible since there is an availability of candidates that do not require further drug development and the pathophysiological involvement can affect one of the axes of direct lung injury. Since 2020, the evidence strongly suggests that the levels of IL-6 correlate with viral load and prognosis in critically ill patients [132]. It is also associated with the presumption that the particular mode of apoptosis in the SARS-CoV-2 infection is pyroptosis, explaining the massive release of IL-1β, IL-2, IL-6, TNF-α, MCP1 and the attrition of CD4 and CD8 T cells [133].

In the initial search, we identified 3051 results of which 43 were selected through the inclusion criteria and relevance; three particular systematic reviews are highlighted amongst the available data. Lan et. al, developed research regarding the effects of tocilizumab in either mortality, intensive care unit admission, or the requirement of mechanical ventilation. They managed to analyze seven studies from a selection of 358 studies, after database research and filtering through inclusion criteria [134]. In their results, they included the studies of Capra et al., Colaneri et al., Klopfenstein et al., Quartuccio et al., Ramaswamy et al., Roumier et al., and Wadud et al. quoted in the Table 1 [26,27,28,29,30,31,32]. According to presented data, the reported studies could not conclude a risk reduction in the overall intervention with tocilizumab, independent of the dose. Taking into account that most of the studies were retrospective in nature, the overall mortality rate for patients with tocilizumab ranged from 3.2% to 38.6% with considerable heterogeneity in the data. Understanding that the authors considered a mean mortality of 24.1% in the control groups, the pooled result did not achieve statistical significance regardless of a pre-established threshold of *p* = 0.1.

Cortegiani et al. performed a similar review including records regarding the use of tocilizumab in viral pneumonia caused by SARS-CoV-2 or sepsis without any restriction in language or methodology. They identified 2071 articles from which 31 were selected according to relevance [135]. Considering the amount of evidence, we will refer to the overall analysis of the database included in this study; the details of the studies can be found in the adjunct table. Summing all the population included in all the clinical data, 5776 patients were analyzed in this review; regarding the characteristics of the studies, the first thing to mention is the fact that 14 studies did not have a comparator, making the quoted results a descriptive outcome [32,33,34,35,36,37,38,39,40,41,42,43,44,45,46,47,48,49,50,51,52,53,136]. Of the remaining 16 studies, 14 suggested tocilizumab improved outcomes related to mortality/ICU admission, nevertheless, some of the quoted studies revealed effect disappearance in the adjusted analysis, as in the case of Martinez et al. [47]. Not all the differences noticed in the studies achieved statistical significance either. Moreover, it is worth noticing that the studies with the largest samples, ranging from 1221 to 1229 individuals, showed mixed results when considering lethality rates, although the design was not intended for comparison in the case of Perrone et al. [49]. Another noticeable tendency in this review was that the studies with the larger samples included very few patients receiving the IL-6 inhibitor compared to the proportion of patients who did not receive the intervention. Finally, a valuable analysis of Cortegiani et al. added a risk of bias in the mentioned data base using the TheROBINS-I tool (Risk of Bias in Non-randomized Studies of Interventions). This allowed the judgement 13 of the studies with a comparator as being poor quality [135].

Khan included a broader perspective regarding the intervention on this pharmacological target they included the aforementioned studies sample but also managed to include the few studies performed with other drugs that attack this same axis [137]. In this review we did not manage to find other studies than those cited in this article with Siltuximab *n* = 1 nor Sarilumab *n* = 3 [71,72,73,74]. Concerning the results, these studies show point estimators that favors the biologic with the same characteristics found in previous studies: observational cohorts with a disproportioned population comparing intervention vs. assigned controls, given the limitations of compassionate use. Two of the Sarilumab studies are descriptive of mortality and most of them have small samples. The Gordon et al. study reflects the tendency with a total population sample *n* = 803 with 353 patients assigned to tocilizumab, 48 to sarilumab and 402 to control [73].

Finally, in our research we found that a great deal of the available evidence was addressed in the previous systematic reviews, however, new evidence has emerged since then. Regarding these other studies, it is noteworthy that evidence is beginning to accumulate with prospective clinical trials, some of them randomized. Sabbatinelli et al., Hermine et al., Salvarani et al., Malekzadeh et al., Dastan et al. and Rodríguez-Molinero et al. have all addressed the question of Interleukin 6 inhibitor and the outcomes on severe or critical COVID-19 patients [54,55,56,57,58,59]; amongst them two studies found clinical results that favors the intervention groups with Tocilizumab, while two are descriptive with no comparator and two showed no difference with the intervention.

### 2.2. Interleukin 1 Inhibitors

Having addressed (IL-6) as one of the important mediators in direct lung injury we cannot forget interleukin 1 (IL-1) as one of the principal actors in the same axis mediating the pyroptosis process mentioned before, even more, considering its similitudes with the macrophage activation syndrome that complicates bacterial sepsis. Data in this environment are partially encouraging [138].

Concerning the data obtained in the initial search, we got 508 hits with the quoted search terms and selected 18 articles after the comprehensive evaluation of the inclusion criteria. The first record to be highlighted is a meta-analysis performed by Kyriazopoulou et al., recording the available data in the use of Anakinra. The aggregate data showed a pooled population of 1185 patients from nine selected studies, with a preliminary search of 209 articles [139]. Of these studies, most of them used either prospective or retrospective observational cohort methodology. The first thing to notice is the consistency of the data with point estimators favoring the intervention with Anakinra, witnessing less objective heterogeneity in the data than that observed in the interventions with the (IL-6) inhibitor, compared with the study of Lan et al. [134]. Nevertheless, from the cited studies, some of them do not reach statistical significance, i.e., Balkhair et al., Kooistra et al., and The CORIMUNO19 Collaborative group [77,81,83]. Furthermore, is worth noticing that even if the overall effect is favoring the biologic, the magnitude of the effect is moderately variable [75,76,78,79,80,82]. The pooled data used in the systematic review of Kyriazopoulou et al. finally estimates an odds ratio (OR) for mortality of 0.37 (95% CI 0.27–0.51; I² 31%) without signs of publication bias in the forest plot [139].

Out of the scope of the aforementioned systematic review, we selected several other studies; one study only presented descriptive results with a small sample in a retrospective manner [85], the rest of them presented association measures regarding death-related endpoints. In these observational studies, we see the same phenomena in the association estimators favoring the use of anakinra, emphasizing that two of them did not achieve statistical significance [84,86]. The last one revealed a significant odds ratio: 3.2 for the use of Anakinra as a survival predictor [88]. It is necessary to address the only other randomized trial made by Kyriazopoulou et al. that is not recorded in their previous metanalysis [87]. This clinical trial preselected severe pneumonia SARS-CoV-2 patients according to soluble urokinase plasminogen receptor plasma levels and randomized (double blinded) for standard care group and Anakinra intervention. The results were deemed significant with a sample of 606 and a risk of death at day 28 hazard ratio = 0.45, 95% CI 0.21–0.98, *p* = 0.045.

Lastly, regarding other less popular IL-1 inhibitors, no studies were found using rilonacept and four studies were selected with the use of Canakinumab. Three of them were observational with descriptive outcomes. Landi et al., described the overall survival rate with no comparator [90], while Katia et al. described reduction in oxygen consumption compared with standard treatment, and Generali et al. referred to a survival rate comparison [91,93]. Although the raw proportion of survival and the reduction in oxygen consumption is statistically significant, the dosage used on interventions are very variable and the samples are relatively small. This leads us to the final piece of evidence in this matter with the only randomized trial found in the effect of Canakinumab and mortality/clinical deterioration measures; Caricchio et al., reported a non-significant mortality risk reduction with Canakinumab with an odds ratio of 0.67 (95%CI, 0.30 to 1.50) regardless of a population sample of 454 patients [92].

### 2.3. Interferons

The value of interferons intertwining with the pathology of the lung injury in SARS-CoV-2 infection radiate from classical signaling pathways described for the most well-defined type I interferons (IFNs). From the known variety, IFNα and IFNβ are the most studied, describing functions in cell antimicrobial states through limiting the spread of infectious pathogens (particularly true for viruses). They interact with the innate immune system, modulating the production of cytokines, promoting antigen presentation and natural killer cell functions while restraining pro-inflammatory pathways. They interact with the adaptive immune system by promoting the development of antigen-specific T and B cell responses, deriving in immunological memory [140]. It is of particular interest the fact that IFNs interact with the JAK 1 axis to reach specific genome sequences for transcription, since this pathway encodes in several types of proteins that restrain pathogens via the inhibition of viral transcription, translation and replication, the degradation of viral nucleic acids, and the alteration of cellular lipid metabolism [141].

In this review, we encountered 206 articles in the preliminary search with a selection of nine records for analysis. The aforementioned Walz et al., included several interferon studies in his analysis of the clinical relevance of JAK inhibitors; some of the used records were specific of pediatric populations or were epidemiological descriptions of the cured patient’s data that deemed to be out of the scope of this review [142]. Still, of the remaining data we found five articles related to group 1 IFNs to be relevant, three observational studies, and two clinical trials [94,95,96,97,98]. Whereas Monfared et al., performed a clinical trial with mortality primary end point, Hung et al. described nasopharyngeal swabs negativization as a surrogate outcome of the resolution of the disease. Both trials favored the use of IFNs in these circumstances given the significance of the differences [94,96]. In the analysis of Walz, the pooled data also supported the fact that interferon reduced the mortality probability (OR, 0.19; 95% CI, 0.04–0.85); *p* = 0.03, *n* = 1906. This, including the other observational data regardless of the descriptive nature of the incidences in these records, is without standardization or control in the disease stage of the intervention, nor the regimes of dosage among centers.

Beyond the noted bibliography, we found only three other studies that complied with the inclusion criteria and were not addressed in other meta-analysis or reviews. Two of the studies referred to group 1 IFNs and one study addressed group 3 IFNs. Zhou et al. described the clearance of real time polymerase chain reaction (RT-PCR) for SARS-CoV-2 as a surrogate of disease improvement to prevent severe pneumonia. They found accelerated viral clearance from the upper respiratory tract in patients who received IFN-a2b treatment (20.4 days, *p* = 0.002), with a mean difference of 7 days with control group [99]. Rahmani et al., completed a randomized clinical trial with a sample of 80 patients considering the mortality outcome as a secondary outcome. The time to clinical improvement was the primary one depicting significant differences HR = 2.30; 95% CI: 1.33–3.39 for a mean difference in two days for resolution [100]. Finally, the only study to portray the effects of another group of interferon in the COVID-19 patients was the one performed by Feld and colleagues. The decline in SARS-CoV-2 RNA was the main outcome, reporting greater reduction in those treated with peginterferon lambda than placebo from day 3 onwards, with a difference of 2.42 log copies per mL at day 7 (*p* = 0.0041) [101].

### 2.4. Mesenchymal Stem Cells

Mesenchymal stem cells are also known as mesenchymal stromal cells. The use of these cells is widely known in certain inflammatory diseases, and also as part of allogenic adoptive transfer therapy and even in graft vs host disease. This might be related to their properties of tissue repair and low immunogenicity. These cells tend to present surface markers, such as CD44, CD90, and CD105, but they are also characterized by the absence of hematopoietic markers, such as CD34, CD45, and HLA-DR. Those characteristics have consequences in cell recognition and may contribute to the anti-inflammatory properties [143].

On the other hand, even if we cannot pinpoint the exact interaction of this pharmacological intervention in the context of SARS-CoV-2 infection, and even if we think of this rationale as insufficient, there is already evidence of its use on other viral-driven lung injuries, such as A/H5N1 acute lung injury [144]. From the available preliminary data, we managed to find 105 articles from which we selected 7 according to the inclusion criteria.

Amongst the selected studies, we managed to find a single meta-analysis. Qu et al., reviewed the available data concerning the use of mesenchymal stromal cells, regardless of the origin (marrow, adipose tissue, or umbilical cord), and evaluated the impact on mortality on adults with acute respiratory distress syndrome (ARDS). They encompass several literatures that addresses ARDS, however, only a single bibliography was related directly with COVID-19 patients. They use indirect evidence to analyze the plausibility of use in critically ill COVID-19 patients. Even more, some of the records used in the review reference to case reports or series of cases. Despite this, it is worth evaluating the conclusions of the pooled data: regarding the secondary outcome of mortality rate, the data seemed to favor treatment with mesenchymal cells without achieving significance: OR 0.63, 95% confidence interval 0.21–1.93. The primary outcome was safety related without reporting any serious adverse events [102].

Regarding the other selected records, only two were observational studies and four of the registries were clinical trials, some of them with randomization and masking. Overall, the studies in this topic tend to have the smallest of samples compared with the above-mentioned pharmacological targets. The consequent analysis derives into mostly descriptive outcomes, regardless of methodology. The incidence of mortality and related outcomes is limited in the small samples. There are studies that in spite of having placebo group as control, did not present a single fatality in either group. All these factors were taken into account in the study design, since most of the outcomes related to either radiological evolution, biomarker evolution, or pulmonary function tests after a predetermined time lapse [103,104,105,106,107]. The characteristics of the studies can be found in the adjunct table.

Lastly, we must highlight a mesenchymal stem cell derived compound used in a single clinical trial performed by Sengupta et al. In this trial, the authors attempted to use exosomes derived from bone marrow mesenchymal stem cells as immunomodulatory mediators that could avoid the possibility of infusional reactions and allergic responses [108]. The limitations of the study are the same as in the cluster of records mentioned above, nevertheless it opens the possibility to another method of implementing this pharmacological target.

### 2.5. Anti-Spike Monoclonal Antibodies

SARS-CoV-2 has four main structural proteins: spike (S), envelope, membrane, and nucleocapsid, being the S protein responsible for receptor attachment and membrane fusion, facilitating viral entry into host cells by binding to angiotensin-converting enzyme 2 (ACE2) receptors [145]. Monoclonal antibodies can help neutralize the virus in infected patients and are used as a passive immunotherapy [146].

The most studied anti spike monoclonal antibodies, based on the findings of this study, are Bamlanivimab (monotherapy) [109] and Bamlanivimab plus Etesevimab [110,111,112], followed by sotrovimab [127,128,129,130,131] and Casirivimab plus imdevimab [124,125,126]. Bamlanivimab/etesevimab has shown positive outcomes in mortality, hospitalization rate, and progression of the disease prevention, as well as Casirivimab/imdevimab did. However, Sotrovimab showed non-concluding results; observational studies demonstrated reduction in hospitalization rate and disease progression, while clinical trials compared to the placebo did not show improvements of clinical outcomes among adults hospitalised with COVID-19. There are no ongoing clinical trials for Bebtelovimab and Tixagevimab/cilgavimab, and the evidence available is limited to evauated evidence regarding the outcomes evaluated in this study.

## 3. Discussion

The use of biologicals in the context of COVID-19 implies a deep understanding of the physiopathological pathways of the infection to address a more directed axis, hoping for new alternatives of management to prevent severe or advanced phases of the illness. However, even if we understand the biological plausibility in each scenario of proposed interventions, we must consider the principle that guides epidemiological studies to endorse interventions. This principle is mainly directed to the degree of certainty that the evidence allows. In a general approach to the complied data in this review, we must stress the common findings in the limitations that these studies share, regardless of the pharmacological target.

First, is the methodological consistency. In this aspect the studies show great variability in their design. We are not referring to the nature of the study itself but the fact that throughout the evolution of the pandemic what is considered standard care changes continuously. If we evaluated what entails standard care in the earliest publications, of each target, we would find that the concomitant use of antivirals, such as lopinavir/ritonavir and the use of Hydroxychloroquine, were considered as standard care. Even if we argue that both control groups and intervention groups were submitted to the same variables, the risk of confounders is there, since we cannot always tell or predict interaction pathways. The multivariate regressions employed in most of the non-descriptive studies can stratify and eliminate some of this burden, however, the standard care in the most recent studies do not entail the same co-interventions.

In this line of thought, we also encounter the difficulty of controlling consistency with over-added variables, according to the selected population in each study, since not every single one performs regressions models. The fact that most of the studies start with a population with severe phase to hyperinflammatory phase, implies that not only are more interventions added as part of the standard care but the fact that dealing with these populations gives different startup lines with great variability in prognosis factors that must be either analyzed or controlled per protocol. The sheer amount of possible prognosis markers and scales can explain in some part the heterogeneity in the cited review studies, as seen in the different conclusions between Lan et. al and Cortegiani et al. [134,135], regarding Tocilizumab.

A second broad point is the inherent limitations in each methodological design. Although observational, cohorts’ studies can evaluate multiple outcomes simultaneously and establish a causal degree of certainty; the control over the multiple variables that can influence the outcome is limited, in contrast to experimental designs. This may sound as an apparent truth, but the volume of observational studies amongst the total of the data extracted may raise some eyebrows regarding of the magnitude of the possibility of unidentified confounding bias. Of course, considering the ethical reservations in the case of a pandemic, this type of study would be popular at the start of the spread since it does not require experiments with an intervention with a preselected population of intervention. Nevertheless, we cannot ignore the strains it poses on the validity of conclusions.

As a third point we must stress the importance of the variable sample size amongst the studies. Even regarding targets with a huge number of studies, such as Il 6, most of the studies have very small sample sizes individually. This can limit the possibility of reaching conclusions that can be extrapolated outside the study environment. Being aware of this might favor methodological designs that prefer surrogate outcomes as biomarkers, pulmonary function tests, radiological improvement, or PCR clearance. These surrogate markers obviously limit the possibility of the wide endorsement use of these pharmacological interventions.

Not only do the sample sizes tend to be small, but also the context of compassionate use determines a disproportionate number of patients in either control groups or intervention groups compared to their counterpart. Many of the cited studies were affected since the view of compassionate use can change in each institution. In some cases, the treatment patients were too few compared to the number of controls even in large samples. In other cases, the number of controls were insufficient as the center where the studies were performed had already implemented the intervention as hospital protocol.

A third point to be addressed is the large amount of evidence that submits pure incidence descriptive outcomes. This type of evidence is valuable to support the notion of the need for randomized trials with larger population samples; however, considering the development of the pandemic with a still relevant number of new deaths, we cannot endorse pharmacological intervention prospects with the analyzed data as a widespread practice. Furthermore, methodological standardization is needed regarding the variability of treatment regimens that differ at each center in each intervention group.

## 4. Materials and Methods

### 4.1. Search Eligibility Criteria and Search Strategy

We performed a systematic review of the literature concerning the use of biological drugs in the context of patients infected with SARS-CoV-2, according to the Preferred Reporting Items for Systematic Review and Meta-Analysis (PRISMA) statement [147]. The systematic review protocol was registered in PROSPERO with the number CRD42022317998. The PICO components sought information regarding adult patients with confirmed SARS-CoV-2 infection, preferring severe or advanced stage of compromise. The selected intervention was the use of biologics, according to the regulatory definition adopted by the “U.S. Food and drug administration” (FDA) prior to the modification “Consolidated Appropriations Act, 2020” that was implemented on 20 December 2019. This was a modification to the norm contemplated in the act: “Biologics Price Competition and Innovation Act of 2009 (BPCI Act)” implemented that year. We chose this definition, taking into account that is aligned with the objective of analyzing the therapies with greater specificity that can bring a benefit to the critically ill patient with COVID-19. Considering that the modification of the end of 2019 allows the inclusion of any chemically synthesized polypeptide and not exclusively of synthesis mediated by cells, tissues, or microorganisms.

Regarding the outcomes for the search, we prioritized any record depicting overall mortality due to SARS-CoV-2 and fatality rates regardless of the nature of primary or secondary outcomes. We also considered time to discharge, risk of mechanical ventilation, and surrogate biomarkers of efficacy.

We performed a search of the relevant bibliographic references through the following databases: Cochrane, Embase, Pubmed, Medline, medrxiv.org, and Google scholar. The search was performed with the following mesh terms: “COVID-19”, “SARS-CoV-2”, “Bio-logical Products”, “Interleukin 6 Receptor Antagonist Protein”, “Interleukin 1 Receptor Antagonist Protein”, “Mesenchymal Stem Cells”, “Mesenchymal Stem Cell Transplantation”, and “anti-spike monoclonal antibody”. We used these terms as exact phrases and a combination of subject headings according to databases syntax. We also performed a search with the most relevant drug names as mesh terms to complement the preliminary findings with the following terms: “Tocilizumab”, “Siltuximab”, “sarilumab”, “Anakinra”, “Canakinumab”, “Interferons” and “Mesenchymal Stem Cells”, “Bamlanivimab plus Etesevimab”, “Casirivimab/imdevimab”, “Bamlanivimab or Casirivimab/imdevimab”, and “Sotrovimab”. The recorded data were also expanded through the relevant references of selected literature on first search. No restriction in language was applied, and the research was performed from its inception until 11 January 2022 (Supplementary Information S1: Search constructs).

### 4.2. Study Selection, and Data Extraction

Once the search was carried out (M.A.), two independent researchers made a preliminary selection of the studies (H.O. and R-H.B). The selection was based on the titles and abstracts, considering the inclusion and exclusion criteria. If the researchers for the selection of a publication reached no consensus, the decision rested in the criteria of a third evaluator.

In a second stage, we applied the following inclusion criteria: full text studies, review articles, observational studies, meta-analysis, or clinical trials investigating the use of a biological drugs, with the intention of reducing mortality in patients infected by SARS-CoV-2; studies investigating the use of a biological drug with the intention of reducing the stay in the intensive care unit; studies investigating efficacy biomarker outcomes in severe or advanced COVID-19 patients.

We also applied the following exclusion criteria: studies that do not use biological drugs; studies that refer exclusively to anticoagulation methods as an exclusive intervention, even if it is done with drugs that are included in the biological category; studies related to vaccination, even if it is done with drugs that are included in the biological category; case report studies or series of cases studies and studies performed in other populations outside adults.

Finally, we performed a descriptive analysis of the literature found in the research and synthetized in the adjunct table (Table 1). For the review of articles in full text, the following information was taken into account: type of study, drug, therapeutic target, sample size (n), dose, and clinical outcome. All the papers found were collected in RAYYAN^®^. EndNote X9 has been used to keep track of references. In this table, we included all the studies except meta-analysis since we chose to list the included studies in each compilation article as raw data. The meta-analysis was referenced and described in the result section in each target. In addition, to avoid publication bias we performed an additional search of unpublished and gray literature in the specified databases for this purpose, such as medrxiv.org and Google scholar.

For observational case-control and cohort studies, Risk Of Bias In Non-Randomized Studies—of Interventions (ROBINS-I) have been used. For clinical trials, the Cochrane risk-of-bias tool for randomized trials (RoB 2) have been used. These tools were applied by two researchers independently. Discrepancies have been resolved by consensus. Studies were stratified according to quality (high risk of bias and low risk of bias), to perform a sensitivity analysis.

## 5. Conclusions

Finally, in conclusion of each case:

Il 6 inhibitors: This pharmacological target has the most amount of accumulated evidence available. We cannot ignore the fact that even with all the limitations mentioned before, most of the point estimators regarding disease resolution, mortality, and mechanical ventilation used tend to favor the intervention in this target. No generalization can be made regarding the use of these pharmacological alternatives since the heterogeneity of the data is high with several studies without statistical significance and a fair number of studies that show no difference with the intervention. We encourage more data recollection with randomized clinical trials, with larger samples, and controlling prognosis factors (i.e., with tools, such as the Charlson score index). The standardization of treatment regimens is needed to accumulate consistent data.

Il 1 inhibitors: The compiled data shows less heterogeneity compared with the Il 6 inhibitors. Most of the point estimators favor this pharmacological group, without overlooking the fact that some of the data are not statistically significant. The number of records and the small samples suggest the need of larger randomized trials, despite the encouraging results. The standardization of treatment regimens is needed to accumulate consistent data.

Interferons: In this group most of the estimators related to death or disease deterioration showed good responses to the intervention, nevertheless, we must stress that half of the data use surrogate or descriptive outcomes and the availability of records within the criteria gives a very small sample. Regardless of the methodology, more data are needed to conclude in this target.

Mesenchymal stem cells: This biological has less data available regarding its efficacy with the studies with the smallest of samples. The descriptive nature of biomarkers as surrogate primary endpoints is widespread amongst the studies. We speculate that the availability and logistical challenges in this matter may limit the number of studies to be performed in the future. Furthermore, even if the results reflected encouraging data the possibility of widespread use in certain countries may limit its implementation.

Anti-spike antibodies: In the case of anti-spike antibodies, the improvement in clinical outcomes in patients with COVID-19 is obtained in seronegative patients. Serological tests are decisive, since otherwise the benefit of these antibodies would be very low. Many questions remain to be answered with the use of anti-spike antibodies in the prevention of clinical outcomes in this type of patient and the interaction of the antibodies with the immune response produced by the vaccines against COVID-19. There is still a need to collect information on the safety and efficacy of these anti-spike antibodies and to have evidence of the influence of virus variants on the clinical response of these antibodies.

## Figures and Tables

**Table 1:** Biotherapeutics in COVID-19 patients.

Drug	Therapeutic Target	*n*	Study Type	Dose	Clinical Outcome	Ref.
Tocilizumab	Il 6	85	Retrospective observational study	400 mg i.v. once (*n* = 33), 324 mg s.c. once (*n* = 27), 800 mg i.v. (*n* = 2)	Survival rate increase favoring tocilizumab hazard ratio for death: 0.035; 95% confidence interval [CI], 0.004 to 0.347; *p* = 0.004	[26]
Tocilizumab	Il 6	112	Retrospective observational study	8 mg/kg i.v. and repeated after 12 h (*n* = 21)	ICU admission and mortality favors tocilizumab OR 0.78; 95% CI between 0.06 and 9.34; *p* = 0.84	[27]
Tocilizumab	Il 6	45	Retrospective case–control study	1 or 2 doses (*n* = 20)	Combined primary endpoint (death and/or ICU admission) was higher in the control group than in the Tocilizumab group (72% vs. 25%, *p* = 0.002)	[28]
Tocilizumab	Il 6	111	Retrospective observational study	8 mg/kg i.v. once (*n* = 42)	Fatality rate and levels of inflammatory markers increase in tocilizumab group 4 of 42 cases died with no fatalities in standard care group	[29]
Tocilizumab	Il 6	86	Retrospective case–control study	400 mg fixed dose or 8 mg/kg (*n* = 21) once or twice	Death rates decrease in tocilizumab group RR 0.472; 95% CI 0.449–0.497	[30]
Tocilizumab	Il 6	59	Retrospective case–control study	8 mg/kg at discretion of the treating physicians,	Death, invasive ventilation reduction in tocilizumab group OR: 0.25 95%CI [0.05–0.95], *p* = 0.04	[31]
Tocilizumab	Il 6	94	Retrospective case–control study	N/A (*n* = 44)	Survival rate in tocilizumab group 61.36% versus 48% in the control group, *p* < 0.00001	[32]
Tocilizumab	Il 6	25	Retrospective observational study	Median total dose 5.7 mg/kg	36% of patients were discharged alive from ICU by day 14 with no comparator	[33]
Tocilizumab	Il 6	65	Prospective observational study	400 mg fixed dose and 24-h 400 mg depending on clinical deterioration	At day 28 (16%) of the tocilizumab group died, compared to 33% of standard treatment group (*p* = 0.150).	[34]
Tocilizumab	Il 6	544	Multicentered retrospective observational study	Tocilizumab 8 mg/kg (up to 800 mg) twice	Hazard ratio of death/mechanical ventilation favors tocilizumab adjusted (hazard ratio 0.61, 95% CI 0.40–0.92; *p* = 0.020)	[35]
Tocilizumab	Il 6	51	Retrospective observational study	Tocilizumab 8 mg/kg and received (up to 400 mg)	Death/clinical improvement at 21 days in treated vs. Control favors control 76.5% (95% CI: 57.3–95.6) vs. 79.4% (95% CI: 56.0–100)	[36]
Tocilizumab	Il 6	15	Retrospective observational study	80−600 mg per time according to clinical worsening	Laboratory data and clinical course with no comparator; 20% of the patients died	[37]
Tocilizumab	Il 6	51	Prospective nonrandomized study	Fixed first dose of 400 mg followed by 400 mg after 12 h	Mortality and clinical course with no comparator 30 day mortality: 27%.	[38]
Tocilizumab	Il 6	153	Prospective observational study	Tocilizumab 8 mg/kg i.v. (up to 800 mg); second dose if elevated body mass	87% survival at day 14 with no comparator	[39]
Tocilizumab	Il 6	63	Prospective observational study	Tocilizumab i.v. 8 mg/kg	11% Mortality at day 14 no comparator	[40]
Tocilizumab	Il 6	100	Prospective observational study	Tocilizumab 8 mg/kg (up to 800 mg) twice	Clinical outcome at day 10: 77% improved or stabilized and 23% worsened no comparator	[41]
Tocilizumab	Il 6	21	Retrospective observational study	Tocilizumab 4–8 mg/kg (up to 800 mg) twice	Mean discharge day 15.1 without comparator	[42]
Tocilizumab	Il 6	89	Retrospective observational study	Tocilizumab 400 mg single dose	Descriptive deaths, mechanical ventilation and discharged with no comparator; 63/72 not mechanically ventilated patients were discharged	[43]
Tocilizumab	Il 6	186	Retrospective observational study	Tocilizumab single dose of 400−600 mg	51 patients were intubated or dead at day 15 with no comparator.	[44]
Tocilizumab	Il 6	547	Retrospective observational study	Tocilizumab: 400 mg some with a second dose of 800 mg	The unadjusted 30 day mortality favored tocilizumab (HR, 0.76, 95% CI, 0.57−1.00)	[45]
Tocilizumab	Il 6	60	Nonrandomized prospective observational study	Tocilizumab 400 mg single dose according to clinical response redosing possibility	Bacterial and fungal infections	[46]
Tocilizumab	Il 6	1229	Multicentered retrospective observational study	Tocilizumab median dose 600 mg, second dosing according to clinical response	Tocilizumab associated with higher risk of death (HR 1.53,95% CI 1.20–1.96, *p* = 0.001)	[47]
Tocilizumab	Il 6	171	Retrospective observational study	Tocilizumab 400 mg/24 for patients with ≤75 kg and 600 mg/24 for patients with >75 kg with second and third dosing according to clinical response	Description of frequency for composite ICU admission or death favoring Tocilizumab (10.3% vs. 195 27.6%, *p* = 0.005)	[48]
Tocilizumab	Il 6	1221	Multicentered phase 2 clinical trial	Tocilizumab 8 mg/kg and second dose according to clinical response	Lower lethality rates at 14 and 30 days (15.6% and 20.0%) among the treated with tocilizumab	[49]
Tocilizumab	Il 6	145	Multicentered retrospective observational study	Tocilizumab 400–800 mg single dose	Descriptive study of mortality with no comparator 43.8% of the population discharged and 29.3% died	[50]
Tocilizumab	Il 6	246	Retrospective observational study	Tocilizumab 400 mg single dose	Composite of all-cause mortality and invasive mechanical ventilation favoring tocilizumab (HR = 0.49 (95% CI 0.3−0.81), *p* = 0.005)	[51]
Tocilizumab	Il 6	82	Prospective and retrospective observational	Tocilizumab 400 mg single dose with second dose according to clinical response; 600 mg if >75 kg	Mortality at 7 days of tocilizumab start; 26.8% of all patients died (no comparator)	[52]
Tocilizumab	Il 6	154	Single center retrospective observational	Tocilizumab 8 mg/kg single dose	Survival probability post intubation favoring tocilizumab in 3 models: model A HR 0.54 (95% CI 0.29, 1.00)	[53]
Tocilizumab	Il 6	29	Single center prospective clinical trial	Tocilizumab 8 mg/kg single dose	Classified as responders or non-responders (secondary analysis described correlation with miR-146a marker) 55.17% of patients where responders	[54]
Tocilizumab	Il 6	130	Prospective multicenter randomized clinical trial	Tocilizumab 8 mg/kg two doses	Risk of mechanical ventilation or death at day 28 favored tocilizumab HR 0.58 (90% CrI, 0.30 to 1.09).	[55]
Tocilizumab	Il 6	126	Prospective randomized clinical trial	Tocilizumab 8 mg/kg up to a maximum of 800 mg	Clinical worsening ratio showed worst outcome in tocilizumab group (risk ratio, 1.05; 95%CI, 0.59–1.86).	[56]
Tocilizumab	Il 6	126	Prospective nonrandomized clinical trial	Tocilizumab 324–486 mg according to body weight single dose	Mortality rates with no comparator: by day 14 of the study, 4.65% (4/86) of severe patients and 50.00% (20/40) of critical patients died.	[57]
Tocilizumab	Il 6	42	Prospective nonrandomized clinical trial	Tocilizumab 400 mg single dose	Mortality rates with no comparator: 35 patients (83.33%) showed clinical improvement by day 28	[58]
Tocilizumab	Il 6	418	Matched cohort study	Tocilizumab up to 3 doses ranging from 400 mg to 600 mg according to clinical evaluation	Inspired oxygen fraction/saturation 48 h post treatment showed no difference, logistic regression didnot show an effect of tocilizumab on mortality (OR 0.99; *p* = 0.990).	[59]
Tocilizumab	IL 6	6837	Meta Analysis	Single IV dose of 8 mg/kg (maximum 800 mg) initially according to clinical evaluation	Reduce in risk of mechanical ventilation at 28–30 days (0.79) and lowers risk of mortality	[60]
Tocilizumab	IL 6	163	Observational cohort study	2 doses of 600 mg on consecutive days	Benefit in the combined treatment with TCZ and CS may have a potential role in reducing mortality	[61]
Tocilizumab	IL 6	567	Meta Analysis	400 mg single dose	Risk of mortality similar in treatment with TCZ alone and comined therapy 0.74 (95% CI: 0.36–1.50)	[62]
Tocilizumab	IL 6	99	Prospective cohort study	400 mg single dose	There were no significant differences in mortality compared to the control group (34% vs. 34%, *p* = 0,98)	[63]
Tocilizumab	IL6	135	Prospective nonrandomized clinical trial	625 mg (mean dose) on 9 consecutive days	No additional survival benefit with TCZ 29% vs. 35% with RR = 0.79 and 95% CI: 0.70–0.89, *p* = 0.01	[64]
Tocilizumab	IL 6	514	Observational retrospective study	400 mg single dose	Significant difference in length of stay of patients with invasive mechanical ventilation (73.1%)	[65]
Tocilizumab	IL 6	100	Phase 2, open-label, randomized study	4 mg/Kg and 8 mg/kg	There was no clear difference between 2 treatment groups in the odds ratio for mortality at day 28	[66]
Tocilizumab	IL 6	23	Retrospective, observational study	400 mg single dose	Rapid clinical improvement with TCZ treatment in the severely ill COVID-19 patients, as opposed to the case in the critically ill patients	[67]
Tocilizumab	IL 6	114	Prospective study	6 mg/kg	At the time point that PaO_2_/FiO_2_ < 200 was observed, improved survival (16.1%) than in the usual care group (32.8%	[68]
Tocilizumab	IL 6	87	Randomised, controlled	6 mg/kg	TCZ associated with a decrease mortality (9.52%) and reduce the invasive mechanical ventilator	[69]
Tocilizumab	IL 6	129	Retrospective cohort study	4–8 mg/kg	In patients with severe or critical COVID-19 was significantly associated with better survival compare with control group (21.6% vs. 42.3% respectively; *p* = 0.015)	[70]
Siltuximab	IL 6	218	Observational cohort study	Siltuximab 2 doses 11 mg/kg	30 day mortality rate favors Siltuximab (HR 0.462, 95% CI 0.221–0.965); *p* = 0.0399).	[71]
Sarilumab	Il 6	28	Observational cohort study	Sarilumab 400 mg single dose	Clinical improvement and lethality rate showed no differences; 61% of patients treated with sarilumab experienced clinical improvement and 7% died	[72]
Sarilumab	Il 6	803	Prospective nonrandomized clinical trial	Sarilumab 400 mg single dose	Descriptive Hospital mortality: 28.0% (98/350) for tocilizumab, 22.2% (10/45) for sarilumab and 35.8% (142/397) for control.	[73]
Sarilumab	Il 6	53	Prospective nonrandomized clinical trial	Sarilumab 400 mg two doses	Descriptive with Sarilumab no comparator; global resolution rate of 83.0% (89.7% in medical wards and 64.3% in ICU) and an overall mortality rate of 5.7%.	[74]
Anakinra	IL 1	22	Observational cohort study	Anakinra 300 mg for two 5 days tapered to 200 mg for 2 days	Descriptive outcomes regarding mechanical ventilation, death, and mean days to discharge (mean days in control group 9.5 and 5 days in Anakinra group)	[75]
Anakinra	IL 1	96	Observational cohort study with historical controls	Anakinra 100 mg twice a day for 72 h, then 100 mg daily for 7 days	Composite endpoint of admission to the ICU for invasive mechanical ventilation or death (HR 0.22 [95% CI 0.10–0.49]; *p* = 0.0002)	[76]
Anakinra	IL 1	153	Randomized control trial	Anakinra 400 mg/day on days 1–3 then 200 mg on day 4, and 100 mg once on day 5	Patient death or need of mechanical ventilation HR 0.97; 90% CrI 0.62 to 1.52	[77]
Anakinra	IL 1	120	Observational cohort study	High dose anakinra non specified	Adjusted risk of death comparing anakinra group with control HR, 0.18, 95% CI, 0.07–0.50, *p = 0*.001,	[78]
Anakinra	IL 1	392	Observational cohort study with historical controls	Anakinra 10 mg/kg/day until clinical benefit	Anakinra group with reduced mortality risk (hazard ratio [HR] 0.450, 95% CI 0.204–0.990, *p* = 0.047)	[79]
Anakinra	IL 1	128	Observational cohort study	Anakinra 100 mg every 8 h for 3 days, with tapering	Mortality reduction favoring anakinra adjusted [HR] = 0.26; *p* < 0.001	[80]
Anakinra	IL 1	21	Observational prospective cohort	Anakinra 300 mg initial dose following 100 mg every 6 h	In the anakinra group, 28 day mortality was 19% vs. 18% in the control group (*p* = 0.87).	[81]
Anakinra	IL 1	130	Observational prospective cohort	Anakinra 100 mg once daily for 10 days	Reduction in 30 day mortality with anakinra (hazard ratio 0.49; 95% CI 0.25–0.97)	[82]
Anakinra	IL 1	69	Observational cohort study with historical controls	Anakinra 100 mg twice daily for 3 days, followed by 100 mg daily for a maximum of 7 days	Hospital death occurred in 13 (29%) of the anakinra-treated group and 11 (46%) of the historical cohort (*p* = 0.082).	[83]
Anakinra	IL 1	93	Observational retrospective Cohort studies	Anakinra minimum use of 100 mg every 12 h (depending on clinical condition and comorbidities)	Survival rate of anakinra vs Tocilizumab: HR 0.46, 95% confidence interval 0.18–1.20	[84]
Anakinra	IL 1	27	Observational retrospective Cohort studies	Anakinra 100 mg every 6 h for at least 3 days, tapering until 7 days	Descriptive of only 9 treated patients with matched cohort of tocilizumab treated patients (9 survivals)	[85]
Anakinra	IL 1	120	Prospective nonrandomized clinical trial	100 mg anakinra daily for 5 days	Patient mortality without significant difference OR of 0.9 (95%CI [0.80–1.01], *p* = 0.067)	[86]
Anakinra	IL 1	606	Multicentered, double blind, randomized, clinical trial	100 mg anakinra daily for 7–10 days	Risk of death at day 28 hazard ratio = 0.45, 95% CI 0.21–0.98, *p* = 0.045	[87]
Anakinra	IL 1	112	Observational cohort study with matched controls	100 mg four times a day, if managed in a regular ward, or 200 mg three times daily if managed in the intensive care unit	Anakinra as a survival predictor at day 28 odds ratio: 3.2; 95% confidence interval, 1.47–7.17	[88]
Anakinra	IL 1	30	Randomized clinical trial	100 mg daily for a median 5 (3–9) days	A significant reduction of 50% in length of hospital stay compared with control (9.50 ± 4.45 vs. 19.00 ± 12.04, *p* = 0.043). A significant reduction in mortality (odds ratio [OR] = 0.32 [95% confidence interval, CI: 0.20–0.51]	[89]
Canakinumab	IL 1	88	Observational prospective cohort	Canakinumab 300 mg single dose	Descriptive outcome with no comparator, overall survival at 1 month was 79.5% (95% CI 68.7–90.3)	[90]
Canakinumab	IL 1	34	Observational prospective cohort	Canakinumab 300 mg single dose	Descriptive oxygen support requirement at 3 time points: reduction in oxygen flow in patients treated with canakinumab (−28.6% at T1 vs. T0 and −40.0% at T2 vs. T1).	[91]
Canakinumab	IL 1	454	Randomized Clinical trial	Canakinumab 450–750 mg single dose	Non-significant mortality risk reduction with Canakinumab odds ratio of 0.67 (95%CI, 0.30 to 1.50)	[92]
Canakinumab	IL 1	48	Prospective case control	Canakinumab 150 mg at day 1 and day 7	Descriptive outcome, survival at 60 days was 90.0% (95% CI 71.9–96.7) in patients treated with canakinumab and 73.3% (95% CI 43.6–89.1)	[93]
Interferon β-1a	interferon β-1a	81	Randomized Clinical trial	12 million IU/mL three times a week for two weeks	Mortality reduction in interferon group at day 28 (OR, 6.65; 95% CI, 1.67 to 26.45) adjusted for confounders.	[94]
Interferon β-1b	interferon β-1b	256	Retrospective cohort	250 mcg on alternate days	Descriptive outcome mortality rate was 24.6% (63/256). 22 patients (20.8%) in the interferon group and 41 (27.3%) in the control group (*p* = 0.229)	[95]
Interferon β-1b	interferon β-1b	127	Randomized Clinical trial	Three doses of 8 million IU on alternate days	Combination group of interferon was independent risk factor for nasopharyngeal swaps negativization HR 4.27 [95% CI 1.82–10.02], *p* = 0.0010; no deaths in either group	[96]
Interferon α-2b	interferon α-2b	814	Multicenter prospective observational study	3 million IU 3 times per week, for 2 weeks	Descriptive outcome: The overall case fatality rate was 2.95% of the infected population. The case fatality rate for patients treated with IFN-a2b was 0.92 (*p* < 0.01)	[97]
Interferon α-2b	interferon α-2b	446	Retrospective multicenter cohort study	Different regimes in each center (non-specified)	IFN therapy is univariably associated with lower mortality (odds ratio [OR] = 0.18, *p* = 0.029)	[98]
Interferon α-2b	interferon α-2b	77	Prospective observational study	5 mIU in inhaled aerosol each day	Accelerated viral clearance from the upper respiratory tract in patients who received IFN-a2b treatment (20.4 days, *p* = 0.002) mean difference of 7 days with control group	[99]
Interferon β-1b	interferon β-1b	80	Randomized clinical trial	250 µg on alternate days	All-cause 28 day mortality was 6.06% and 18.18% in the IFN and control groups, respectively (*p* = 0.12)	[100]
Peginterferon lambda	interferon lambda	60	Randomized Clinical trial	180 mcg single dose	Favors faster viral clearance with pegylated interferon 2.42 log copies per mL at day 7 (*p* =0.0041)	[101]
Mesenchymal stem cells	Mesenchymal stem cells	200	Meta analysis	Variable according to study and type of mesenchymal stem cells	Favor treatment with mesenchymal cells without achieving significance: OR 0.63, 95% confidence interval 0.21–1.93	[102]
Mesenchymal stem cells	Mesenchymal stem cells	10	Nonrandomized pilot clinical trial	1 × 10^6^ cells per kilogram of weight single transplantation	Descriptive outcome favoring treatment group: none of the patients in the mesenchymal stem cell group died	[103]
Mesenchymal stem cells (umbilical cord)	Mesenchymal stem cells	41	Randomized clinical trial	2 × 10^6^ cells per kilogram of weight single transplantation	Descriptive outcome favoring treatment group: none of the patients in the mesenchymal stem cell group died	[104]
Mesenchymal stem cells (umbilical cord)	Mesenchymal stem cells	18	Nonrandomized clinical trial	Three transplantations of 3 × 10^7^ cells per infusion	Descriptive outcome: mechanical ventilation was required in one patient in the treatment group compared with four in the control group	[105]
Mesenchymal stem cells	Mesenchymal stem cells	25	Retrospective observational study	1 × 10^6^ mononuclear cells per kilogram of weight per infusion every 5 days	No differences comparing Mesenchymal cell treatment and placebo group (inflammatory markers surrogate did not show any differences either)	[106]
Mesenchymal stem cells	Mesenchymal stem cells	100	Randomized double blind clinical trial	Three transplantations of 4 × 10^7^ cells per infusion	Lung function in 6 min walking test at day 28 favors mesenchymal cell treatment median 420 m vs. 403 m in control group *p* = 0.057	[107]
Exosomes Derived from Bone Marrow Mesenchymal Stem Cells	Mesenchymal stem cells	27	Prospective nonrandomized cohort study	15 mL intravenous dose of ExoFlo single dose	Descriptive outcome with no comparator with overall survival rate in the study of 83%.	[108]
Bamlanivimab	Spike protein	467	Randomized, double-blind, placebo-controlled, single-dose trial	700 mg (101 patients), 2800 mg (107 patients), or 7000 mg (101 patients)	Descriptive outcome: At day 29, the percentage of patients who were hospitalized with COVID-19 was 1.6% (5 of 309 patients) in the LY-CoV555 group and 6.3% (9 of 143 patients) in the placebo group	[109]
Bamlanivimab plus Etesevimab	Spike protein	452	Randomized, double blinded clin-ical tria	Bamlanivimab and etesevimab, 2800 mg of each given intravenously	Descriptive outcome: By day 29, a total of 11 of 518 patients (2.1%) in the bamlanivimab–etesevimab group had a COVID-19-related hospitalization or death from any cause, as compared with 36 of 517 patients (7.0%) in the placebo group (absolute risk difference, −4.8 percentage points; 95% confidence interval [CI], −7.4 to −2.3; relative risk difference, 70%; *p* < 0.001)	[110]
Bamlanivimab plus Etesevimab	Spike protein	14,461	Meta analysis	Variable according to study	Favor treatment with Bamlanivimab plus Etesevimab; Bmlanivimab may help outpatients to prevent hospitalizationor emergency department visits (RR 0.41, 95%CI 0.29−0.58), reduce ICU admission (RR 0.47, 95%CI 0.23−0.92), and mortality (RR 0.32, 95%CI 0.13−0.77)from the disease. The combination of bamlanivimab and etesevimab may have agreater potential for positive treatment outcomes.	[111]
Bamlanivimab plus Etesevimab	Spike protein	577	Systematic review	2800 mg IV	Bamlanivimab 2800 mg plus etesevimab 2800 mg: significant difference in hospitalizations/emergency department visit versus placebo; absolute risk difference was −4.9% (95% CI: −8.9% to −0.8%; *p* = 0.049)	[112]
Casirivimab/imdevimab	Spike protein	9785	Randomized, double-blind, placebo-controlled clinical trial	8000 mg IV infusion	Favor treatment casirivimab/imdevimab in addition to usual care with 20% reduction in all-cause mortality (rate ratio 0.80; 95% CI 0.70–0.91; *p* = 0.001); 17% lower relative risk of progressing to invasive mechanical ventilation or death (composite endpoint) with casirivimab/imdevimab plus usual care than with usual care alone among seronegative patients not on such ventilation at baseline (30% vs. 37% of patients; relative risk ratio 0.83; 95% CI 0.75–0.92);	[113]
Casirivimab/ imdevimab	Spike protein	2067	Randomized, double-blind, placebo-controlled, phase 3 trial	Subcutaneous dose of 1200 mg	Casirivimab/imdevimab was effective in preventing symptomatic and asymptomatic SARS-CoV-2 infection, a relative risk reduction of 81.4% (odds ratio [OR] 0.17; 95% CI 0.09–0.33; *p* < 0.001).	[114]
Casirivimab/ imdevimab	Spike protein	275	Double-blind, phase 1–3 trial	2.4 g of REGN-COV2, or 8.0 g of REGN-COV2	The REGN-COV2 antibody cocktail reduced viral load, with a greater effect in patients whose immune response had not yet been initiated or who had a high viral load at baseline.	[115]
Casirivimab/ imdevimab	Spike protein	1505	Randomized, double-blind, placebo-controlled, phase 3 trial	1200 mg of REGEN-COV	Subcutaneous REGEN-COV prevented symptomatic COVID-19 and asymptomatic SARS-CoV-2 infection in previously uninfected household contacts of infected persons. Among the participants who became infected, REGEN-COV reduced the duration of symptomatic disease and the duration of a high viral load.	[116]
Casirivimab/ imdevimab	Spike protein	2696	Adaptive trial	2 groups: 2400-mg group and 1200-mg group	EGEN-COV reduced the risk of COVID-19-related hospitalization or death from any cause, and it resolved symptoms and reduced the SARS-CoV-2 viral load more rapidly than placebo	[117]
Casirivimab/ imdevimab	Spike protein	3596	Observational study	N.S.	Descriptive outcome: no significant difference in all-cause and COVID-19-related hospitalization rates between bamlanivimab and casirivimab-imdevimab (adjusted hazard ratios [95% confidence interval], 1.4 [0.9–2.2] and 1.6 [0.8–2.7], respectively).	[118]
Casirivimab/ imdevimab	Spike protein		Systematic review	N.S.	Prevention of COVID-19 progression from asymptomatic to symptomatic disease in early SARS-CoV-2 infection; patients with mild-to-moderate COVID-19 exhibited reduced hospital utilization after receiving REGN-COV2 treatment within a few days of symptom onset, and a low-dose REGN-COV2 infusion has been shown to improve COVID-19 symptoms; Subcutaneously injected REGN-COV2 prevented SARS-CoV-2 infection and the presence of COVID-19 symptoms in high-risk individuals who had close contact with SARS-CoV-2-infected persons.	[119]
Casirivimab/ imdevimab	Spike protein	2067	Review	Subcutaneous injection of 1200 mg REGEN-COV	The combination of monoclonal antibodies significantly reduced the incidence of symptomatic and asymptomatic SARS-CoV-2 infection, viral load, duration of symptomatic disease and the duration of a high viral load	[120]
Bamlanivimab or Casirivimab/ imdevimab	Spike protein	707	Observational study	N.S.	Patients receiving NmAb infusion had significantly lower hospitalization rates (5.8% vs. 11.4%, *p* < 0.0001), shorter length of stay if hospitalized (mean, 5.2 vs. 7.4 days; *p* = 0.02), and fewer ED visits within 30 days post-index (8.1% vs. 12.3%, *p* = 0.003) than controls. Hospitalization-free survival was significantly longer in NmAb patients compared with controls (*p* < 0.0001). There was a trend towards a lower hospitalization rate among patients who received NmAbs within 2–4 days after symptom onset.	[121]
Bamlanivimab or Casirivimab/	Spike protein	285	Single-center prospective observational cohort study	N.S.	Favoring cocktail group: Assessing all the symptoms, the number of symptomatic individuals on Day 7 was significantly lower in the cocktail group than in the SOC group (23/108 [21.30%] vs. 39/78 [50.0%]; *p* = 0.0001) while the remaining patients in each of the groups recovered completely. (cocktail group: casirivimab/imdevimab; SOC: standard-of-care)	[122]
imdevimab	Spike protein	115	Obervational study	N.S.	Administering monoclonal antibody therapy for high-risk patients with COVID-19 using a regional severity prediction scoring system notably reduced the number of hospitalisations and severe cases	[123]
Casirivimab/ imdevimab	Spike protein	108	Retrospective cohort study	120 mg casirivimab and 120 mg imdevimab	Descriptive outcome: After the treatment, the number of patients with COVID-19-related hospitalization, due to decreased SpO2, was 12, accounting for 11% of the enrolled patients who received REGN-COV2.	[124]
Casirivimab/ imdevimab	Spike protein	165	Observational prospective study	Bamlanivimab (700 mg) com-bined with etesevimab (1400 mg) or casirivimab (1200 mg)combined with imdevimab (1200 mg).	In the Gamma viral strain group, a higher proportion of patients treated with bamlanivimab/etesevimab met the primary endpoint (a composite of hospitalization or death within 30 days from mAbs infusion) compared to those receiving casirivimab/imdevimab (55% vs. 17.4%, *p* = 0.013).	[125]
Casirivimab/ imdevimab	Spike protein	696	Retrospective cohort	One hour infusion of casirivimab (1200-mg dose) and imdevimab (1200-mg dose)	Patients who received casirivimab–imdevimab had significantly lower all-cause hospitalization rates at day 14 (1.3% vs. 3.3%; Absolute Difference: 2.0%; 95% confidence interval (CI): 0.5–3.7%), day 21 (1.3% vs. 4.2%; Absolute Difference: 2.9%; 95% CI: 1.2–4.7%), and day 28 (1.6% vs. 4.8%; Absolute Difference: 3.2%; 95% CI: 1.4–5.1%)	[126]
Sotrovimab	Spike protein	583	Ongoing, multicenter, double-blind, trial	500 mg	A total of (1%) in the sotrovimab group, as compared with 21 patients (7%) in the placebo group, had disease progression leading to hospitalization or death (relative risk reduction, 85%; 97.24% confidence interval, 44 to 96; *p* = 0.002). The clinical progression of COVID-19 at Day 29 in recipients of sotrovimab was reduced by 85% compared with the placebo group (*p* = 0.002)	[127]
Sotrovimab	Spike protein	n/a	Systematic review	N.S.	Treatment with sotrovimab may reduce the number of participants with oxygen requirement (RR 0.11, 95% CI 0.02 to 0.45), hospital admission or death by day 30 (RR 0.14, 95% CI 0.04 to 0.48), grades 3–4 AEs (RR 0.26, 95% CI 0.12 to 0.60), SAEs (RR 0.27, 95% CI 0.12 to 0.63) and may have little or no effect on any grade AEs (RR 0.87, 95% CI 0.66 to 1.16).	[128]
Sotrovimab	Spike protein	546	Multinational, double-blind, randomised, placebo-controlled, clinical trial	500 mg	Neither sotrovimab nor BRII-196 plus BRII-198 showed efficacy for improving clinical outcomes among adults hospitalised with COVID-19. At day 5, neither the sotrovimab group nor the BRII-196 plus BRII-198 group had significantly higher odds of more favourable outcomes than the placebo group on either the pulmonary scale (adjusted odds ratio sotrovimab 1.07 [95% CI 0.74-1.56]; BRII-196 plus BRII-198 0.98 [95% CI 0.67–1.43]) or the pulmonary-plus complications scale (sotrovimab 1.08 [0.74–1.58]; BRII-196 plus BRII-198 1.00 [0.68–1.46])	[129]
Sotrovimab	Spike protein	n/a	Systematic review and network meta-analysis	NS	Patients with non-severe disease randomised to antiviral monoclonal antibodies had lower risk of hospitalisation than those who received placebo: sotrovimab (OR 0.17 (0.04 to 0.57); RD −4.8%; low certainty). They did not have an important impact on any other outcome.	[130]
Sotrovimab	Spike protein	10,036	Observational cohort study	NS	Sotrovimab treatment was associated with a 63% decrease in the odds of all-cause hospitalization (raw rate 2.1% versus 5.7%; adjusted OR 0.37, 95% CI 0.19–0.66) and an 89% decrease in the odds of all-cause 28 day mortality (raw rate 0% versus 1.0%; adjustced OR 0.11, 95% CI 0.0–0.79), and may reduce respiratory disease severity among those hospitalized.	[131]

NS: Not specified, IV: Intravenous.

## Data Availability

The data presented in this study are available in article and supplementary material.

## Supplementary Materials

The following supporting information can be downloaded at: https://www.mdpi.com/article/10.3390/ph15070783/s1, S1: Search constructs, Figure S1: Meta-analysis of the studies that determined mortality with Tocilizumab vs standard therapy.
