# Supplementary material for: Biologics in COVID-19 So Far: Systematic Review
Source: Pharmaceuticals (Basel). 2022 Jun 23;15(7):783. doi: 10.3390/ph15070783 (PMC9321859; doi:10.3390/ph15070783)

Supplementary information S1.

Search constructs:

- ("COVID-19"[Mesh] OR "SARS-CoV-2"[Mesh]) AND "Receptors, Interleukin-6/antagonists and inhibitors"[Mesh]
- ("COVID-19"[Mesh] OR "SARS-CoV-2"[Mesh]) AND "tocilizumab" [Supplementary Concept]
- ("COVID-19"[Mesh] OR "SARS-CoV-2"[Mesh]) AND "siltuximab" [Supplementary Concept]
- ("COVID-19"[Mesh] OR "SARS-CoV-2"[Mesh]) AND "sarilumab" [Supplementary Concept]
- ("COVID-19"[Mesh] OR "SARS-CoV-2"[Mesh]) AND "Receptors, Interleukin-1/antagonists and inhibitors"[Mesh]
- ("COVID-19"[Mesh] OR "SARS-CoV-2"[Mesh]) AND "anakinra" [Supplementary Concept]
- ("COVID-19"[Mesh] OR "SARS-CoV-2"[Mesh]) AND "canakinumab" [Supplementary Concept]
- ("COVID-19"[Mesh] OR "SARS-CoV-2"[Mesh]) AND ("Interferons"[Mesh] OR "Interferon Type I"[Mesh] OR "interferon type III" [Supplementary Concept])
- ("COVID-19"[Mesh] OR "SARS-CoV-2"[Mesh]) AND ("Mesenchymal Stem Cell Transplantation"[Mesh] OR "Mesenchymal Stem Cells"[Mesh])
- ("COVID-19"[Mesh] OR "SARS-CoV-2"[Mesh]) AND ("anti-spike antibody"[Mesh] OR "Bamlanivimab"[Mesh] OR "Bamlanivimab plus Etesevimab"[Mesh] OR "Casirivimab/ imdevimab"[Mesh] OR "Bamlanivimab or Casirivimab/imdevimab"[Mesh] OR “Sotrovimab” [Mesh])

((((((pneumonia SARS-coV-2 infection) OR (pneumonic SARS-coV-2 infection)) OR (pneumonic sars-cov 2)) OR (pneumonic)) OR (sars cov-2)) OR (COVID-19)) AND ((((((((((((Mesenchymal Stem Cells) OR (Mesenchymal Stem Cell Transplantation)) OR (Interferon Type I)) OR (Interferons)) OR (canakinumab)) OR (anakinra)) OR (Receptors, Interleukin-1/antagonists and inhibitors)) OR (sarilumab)) OR (siltuximab)) OR (tocilizumab)) OR (Receptors, Interleukin-6/antagonists and inhibitors) OR (anti-spike antibody) OR (Bamlanivimab) OR (Bamlanivimab plus Etesevimab) OR (Casirivimab/ imdevimab) OR (sotrovimab)(AND ((length stay covid) OR (mortality))

Home | About PROSPERO | How to register | Service information Search | My PROSPERO | Logout: **Rosa-Helena Bustos**

Register your review now

Edit your details

You have 1 records

Records that are being assessed

These records have been submitted for publication and are being assessed by the editorial team. You cannot make changes to these records while they are going through the editorial process.

ID	Title	Status	Last edited
317998	Biologics in treatment of Covid-19 infection: systematic review	Not yet registered	16/03/2022

Figure S1. Meta-analysis of the studies that determined mortality with Tocilizumab vs standard therapy

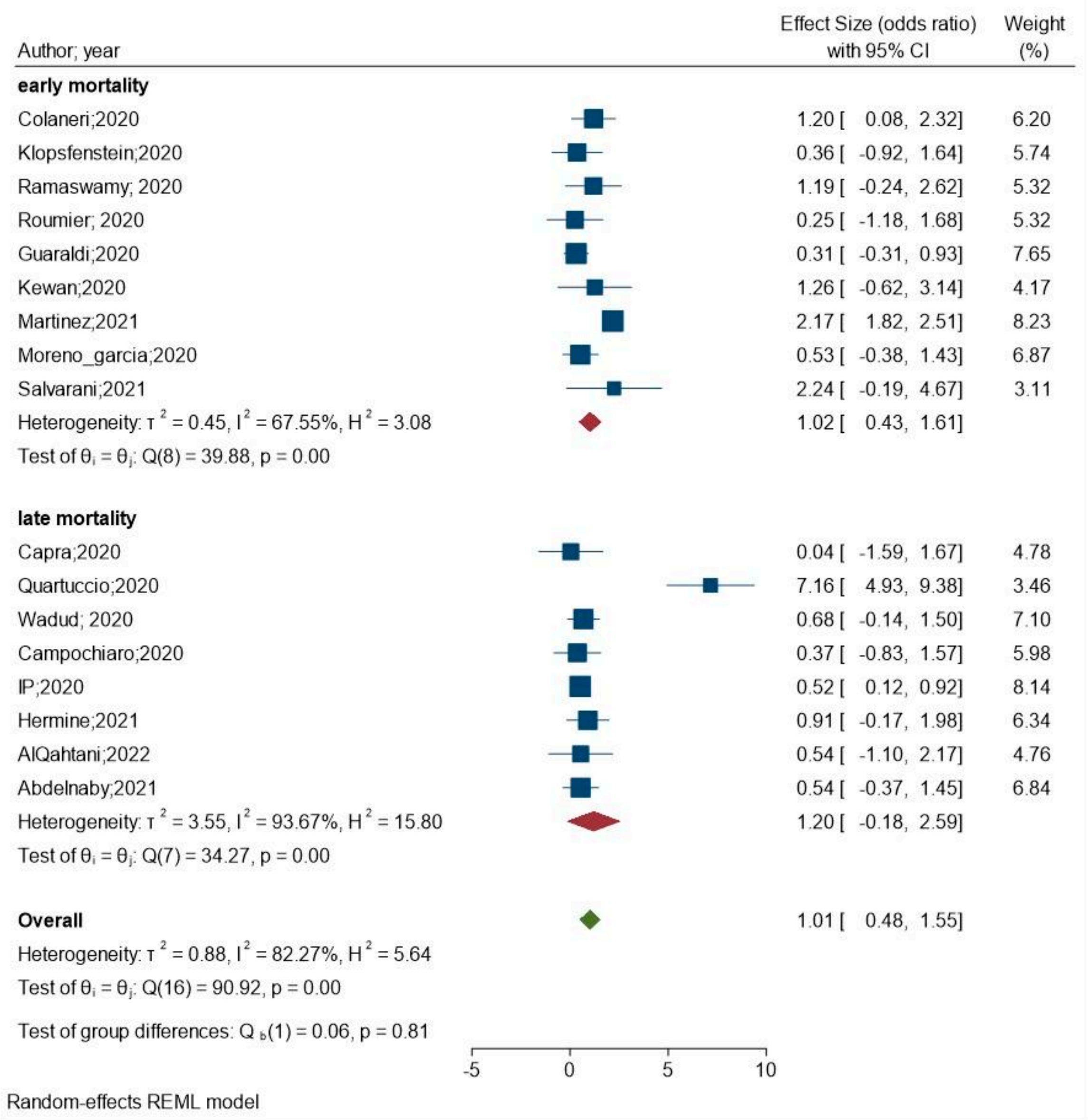

Supplement: Supplementary file 1 [file pharmaceuticals-15-00783-s001.zip › pharmaceuticals-1658962-supplementary.pdf]
